# Aminopeptidase T of M29 Family Acts as A Novel Intracellular Virulence Factor for *Listeria monocytogenes* Infection

**DOI:** 10.1038/srep17370

**Published:** 2015-11-27

**Authors:** Changyong Cheng, Xiaowen Wang, Zhimei Dong, Chunyan Shao, Yongchun Yang, Weihuan Fang, Chun Fang, Hang Wang, Menghua Yang, Lingli Jiang, Xiangyang Zhou, Houhui Song

**Affiliations:** 1 College of Animal Science and Technology, Zhejiang A&F University, 88 Huanchengbei Road, Lin’an, Zhejiang 311300, P. R. China; 2 Zhejiang University Institute of Preventive Veterinary Medicine, 866 Yuhangtang Road, Hangzhou, Zhejiang 310058, P. R. China; 3 Zhoushan Entry-Exit Inspection and Quarantine Bureau, 555 Haijing Road, Zhoushan, Zhejiang 316000, P. R. China

## Abstract

The foodborne pathogen *Listeria monocytogenes* employs a number of virulence determinants including metalloproteases to infect hosts. Here for the first time, we identified an M29 family aminopeptidase T (encoded by *lmo1603*) from *L. monocytogenes* that possesses a typical feature to catalyze the cleavage of amino acids from peptide substrates, with a preference for arginine. The purified recombinant Lmo1603 was activated by Fe^3+^, Zn^2+^ and Mn^2+^, but strongly stimulated by Co^2+^, indicating that Lmo1603 is a cobalt-dependent aminopeptidase. Single mutation at any of the Glu216, Glu281, His308, Tyr315, His327, and Asp329 completely abolished the enzymatic activity of Lmo1603. More importantly, we showed that Lmo1603 was mainly involved in *Listeria* infection, but not required for growth in rich laboratory medium and minimal defined medium. Disruption of Lmo1603 resulted in almost complete attenuation of *Listeria* virulence in a mouse infection model. In addition, we demonstrated that Lmo1603 was mainly localized in the bacterial cytosol and required for invasion and survival inside human epithelial cells and murine macrophages. We conclude that Lmo1603 encodes a functional aminopeptidase T of M29 family, which acts as a novel intracellular virulence factor essential in the successful establishment of *L. monocytogenes* infections in a mouse model.

*Listeria monocytogenes* (*L. monocytogenes*) is a foodborne bacterial pathogen capable of invasion and replication in phagocytic and non-phagocytic cells. This capacity allows it to cross the protective epithelial barriers of the human body and cause severe infection with high mortality, especially in aging populations, pregnant women, infants, and immuno-compromised individuals^1^,^2^,^3^. The ability of *L. monocytogenes* to cause disease in a mammalian host depends upon expression of a number of virulence determinants that enable this pathogen to successfully gain entry into host cells, escape from host cell vacuoles, replicate within the cytosol, and spread to adjacent cells^4^,^5^,^6^. These virulence factors mainly include internalins, listeriolysin O, phospholipases, actin polymerization protein, and, metalloproteases families^5^,^7^.

Aminopeptidases (APs), one of the metalloprotease groups, are ubiquitous enzymes, frequently found in animals, plants and microorganisms. As exopeptidases, APs catalyze the cleavage of free amino acids from peptides. APs are involved in various functions in the cells, such as protein maturation, protein turnover, hydrolysis of regulatory peptides, nitrogen nutrition, and modulation of gene expression, and thus are considered essential enzymes^8^,^9^,^10^. Based on the hierarchical, structure-based classification of the peptidases, APs are divided into clans MF, MG, MH, and MQ in the MEROPS data base (http://merops.sanger.ac.uk)^11^,^12^.

Thermophilic aminopeptidases (AmpT), also named MEROPS family M29, belongs to the clan MQ. The M29 family encompasses aminopeptidase S (AmpS) from *Staphylococcus aureus*^13^, aminopeptidase II (AmpII) from *Bacillus*^14^, aminopeptidase T from *Thermus aquaticus*^15^, PepS aminopeptidase from *Streptococcus pneumoniae*^16^, and their homologues in each member group. Structural information on clan MQ peptidases has so far relied exclusively on the crystal structures of AmpS^13^ and AmpT^17^. In addition, most of the previous studies on peptidases in the M29 family focused on their biochemical properties and biophysical characterizations^18^,^19^,^20^. Despite the emerging roles of aminopeptidases, the members in the M29 family remain poorly understood. Homologues of the M29 family aminopeptidase have also been found in the sequenced genome of *L. monocytogenes* EGD-e by *in silico* analysis^21^. Lmo1603 is predicted to be a member of the M29 family by using the MEROPS database^22^. Little is known of the function of Lmo1603, although it is annotated as an aminopeptidase. Here, we elucidated for the first time the characteristics and functions of Lmo1603 from *L. monocytogenes* by using biochemical and genetic methods as well as biological assays. Lmo1603 encodes a functional aminopeptidase T, which belongs to the aminopeptidase T family and exhibits rather broad substrate specificity of different residues, with a preference for arginine. In addition, we demonstrated that this enzyme is not required for growth in rich or defined medium. More importantly, Lmo1603 is involved in invasion and intracellular survival inside the host cells and required for full virulence in a murine infection model. Therefore, we conclude that Lmo1603 is a novel virulence factor essential for *Listeria* pathogenesis.

## Results

### Lmo1603 is a member of M29 family aminopeptidases

**Figure 1:**
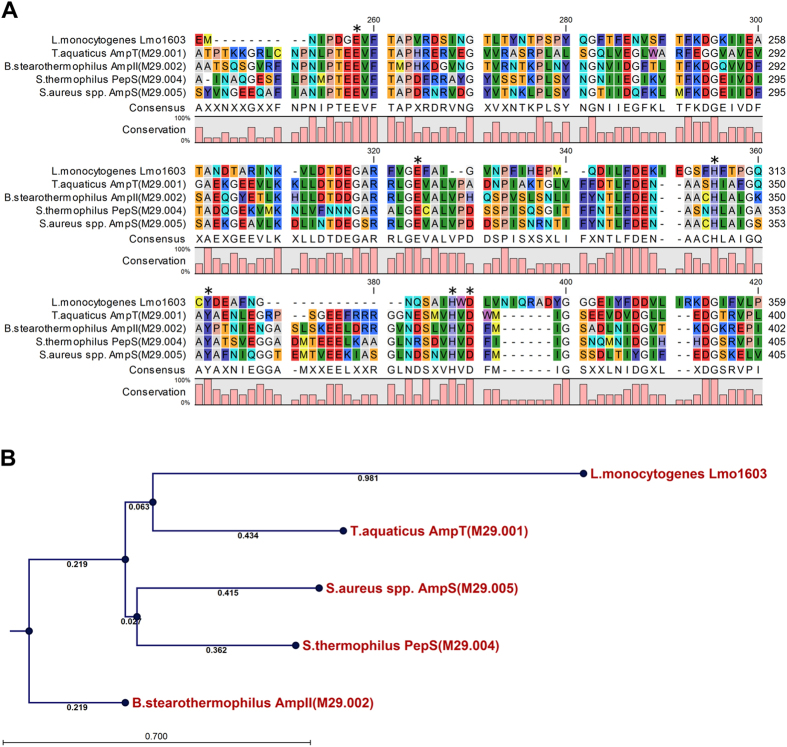
Lmo1603 is a member of M29 family aminopeptidases. (**A**) Amino acid sequence alignment of *L. monocytogenes* putative aminopeptidase T (Lmo1603) against the members of the M29 family from *Thermus aquaticus*, *Bacillus stearothermophilus*, *Staphylococcus aureus* and *Streptococcus thermophilus*. The key amino acid residues denoted with asterisks are involved in enzyme catalytic activity. (**B**) Phylogenetic tree of *L. monocytogenes* putative aminopeptidase T (Lmo1603) and the members of the M29 family. The tree was constructed by the Neighbor-Joining (NJ) program and a bootstrap test of 1000 replicates was used to estimate the confidence of branching patterns, where numbers on internal nodes are the support values.

Based on sequence alignment using the MEROPS database (employing a BLAST search of the database using the full length sequence from *L. monocytogenes*), Lmo1603 is a member of the M29 family aminopeptidases. All enzymes of the M29 family require metal cations for activity and contain a highly conserved catalytic triad (Glu-Glu-His-Tyr-His-Asp), which plays a significant role in catalysis and substrate binding^16^,^17^. Although Lmo1603 shows relatively low amino acid sequence homologies (19.7%–22.1%) to other members of the M29 family, including aminopeptidase T (M29.001), aminopeptidase II (M29.002), PepS aminopeptidase (M29.004) and aminopeptidase S (M29.005)^11^,^12^, the residues of the predicted catalytic site and ligand binding site are 100% conserved between Lmo1603 and the other members of the M29 family (Fig. 1A). This indicates that Glu216, Glu281, His308, Tyr315, His327 and Asp329 are the corresponding active sites of Lmo1603 (Fig. 1A). In addition, phylogenetic analysis revealed that Lmo1603 forms a closer sister branch with aminopeptidase T (AmpT) from *Thermus. aquaticus* (Fig. 1B). Moreover, we modeled the structure of Lmo1603 using the known crystal structures of the members from M29 family as the templates^13^,^16^,^17^ (Figure S1). The predicted protein structure of Lmo1603 has high similarities to the members of M29 family, including *Staphylococcus aureus* AmpS (PDB: 1ZJC)^13^, *Thermus thermophilus* AmpT (PDB: 2AYI)^17^, and *Streptococcus pneumoniae* PepS (PDB: 4ICQ)^16^ (Figure S1). The Lmo1603 dimer has an elongated shape consisting of an N-terminal domain and a C-terminal catalytic domain. The N-domain consists of at least seven helices that are organized around a central, parallel β–sheet, and the C-domain is organized around two β-sheets^13^. Moreover, the putative structure of Lmo1603 contains two cobalt or zinc ions in their active centers with full occupancy. These suggest that *L. monocytogenes lmo1603* encodes an M29 family aminopeptidase T with typical structure arrangements of the peptidase family.

### Lmo1603 is a functional aminopeptidase with a broad substrate specificity

**Figure 2:**
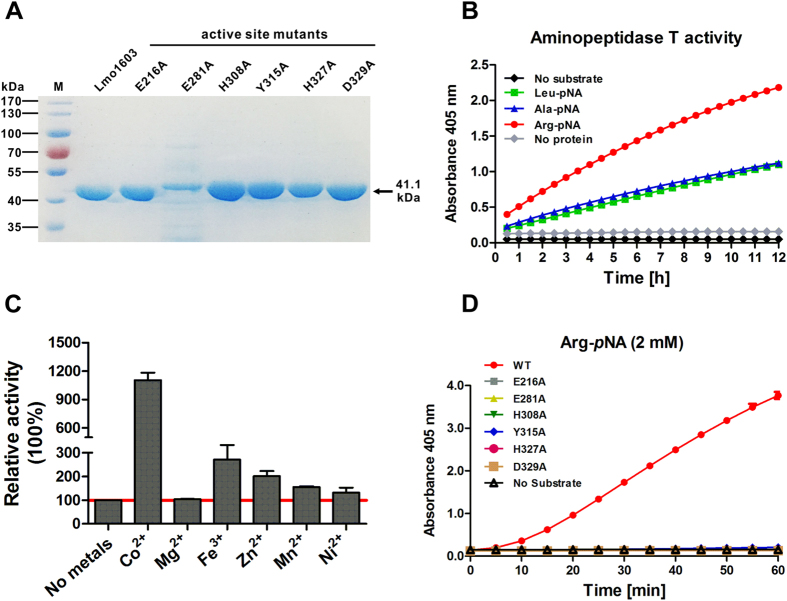
Lmo1603 is a functional aminopeptidase dependent on metal ions. (**A**) SDS-PAGE analysis of the recombinant Lmo1603 and its mutant proteins. The interest proteins are indicated by the arrow; **(B**) Kinetic aminopeptidase activity analysis of Lmo1603 using Arg-*p*NA, Ala-*p*NA or Leu-*p*NA as the substrate in 50 mM Tris-HCl (pH 8.5) containing 2 mM substrate. (**C**) Effects of metal ions (Co^2+^, Mg^2+^, Fe^3+^, Zn^2+^, Mn^2+^ or Ni^2+^, each of 1 mM) on enzymatic activity of Lmo1603. (**D**) Enzymatic activity of wild-type Lmo1603 and its mutant proteins (E216A, E281A, H308A, Y315A, H327A and D329A) using Arg-pNA as the substrate in 50 mM Tris-HCl (pH 8.5) containing 1 mM Co^2+^. Values are expressed as Mean ± SD.

The recombinant Lmo1603 and its mutant proteins in the predicted active sites (E216A, E281A, H308A, Y315A, H327A and D329A) were expressed in *E. coli* and then purified to homogeneity by nickel chelated affinity column chromatography (Fig. 2A). Enzymatic assays were performed by incubation of 5 μM enzyme in the reaction buffer containing 2 mM Arg-*p*NA, Leu-*p*NA or Ala-*p*NA as a substrate and the released *p*-nitroaniline was measured at the indicated time points. As shown in Fig. 2B, the recombinant Lmo1603 was able to degrade all the amino acid-*p*NA substrates to release *p*NA within 12 h. Significantly, Lmo1603 showed the highest activity towards Arg-*p*NA, followed by Leu-*p*NA and Ala-*p*NA (Fig. 2B). These data indicate that *L. monocytogenes lmo1603* encodes a functional M29 family aminopeptidase T^15^.

### Lmo1603 is a cobalt-activated metalloenzyme

Many APs belong to metalloproteases, such as lysine aminopeptidase^23^ and leucine aminopeptidase^24^. As shown in this study, Fe^3+^, Zn^2+^ and Mn^2+^ had moderate activation on this enzyme, with 271%, 200% and 155% relative activity, respectively (Fig. 2C). Interestingly, the enzymatic activity of Lmo1603 was strongly activated by Co^2+^ (1104% relative activity) (Fig. 2C), indicating that Lmo1603 is a cobalt-activated metalloenzyme^25^,^26^.

### Key residues are involved in aminopeptidase activity of Lmo1603

Bioinformatic analysis suggests that Glu216, Glu281, His308, Tyr315, His327 and Asp329 are the corresponding catalytic residues for Lmo1603 (Fig. 1A). To verify these predicted active sites within Lmo1603, site directed mutagenesis targeted at these residues was used to construct mutant proteins. All mutations led to almost complete loss of catalytic ability towards substrate Arg-*p*NA (Fig. 2D), which is consistent with those obtained for other members of M29 family^13^,^16^,^17^.

### Lmo1603 is essential for *L. monocytogenes* virulence in mice infection model, but not required for growth *in vitro*

**Figure 3:**
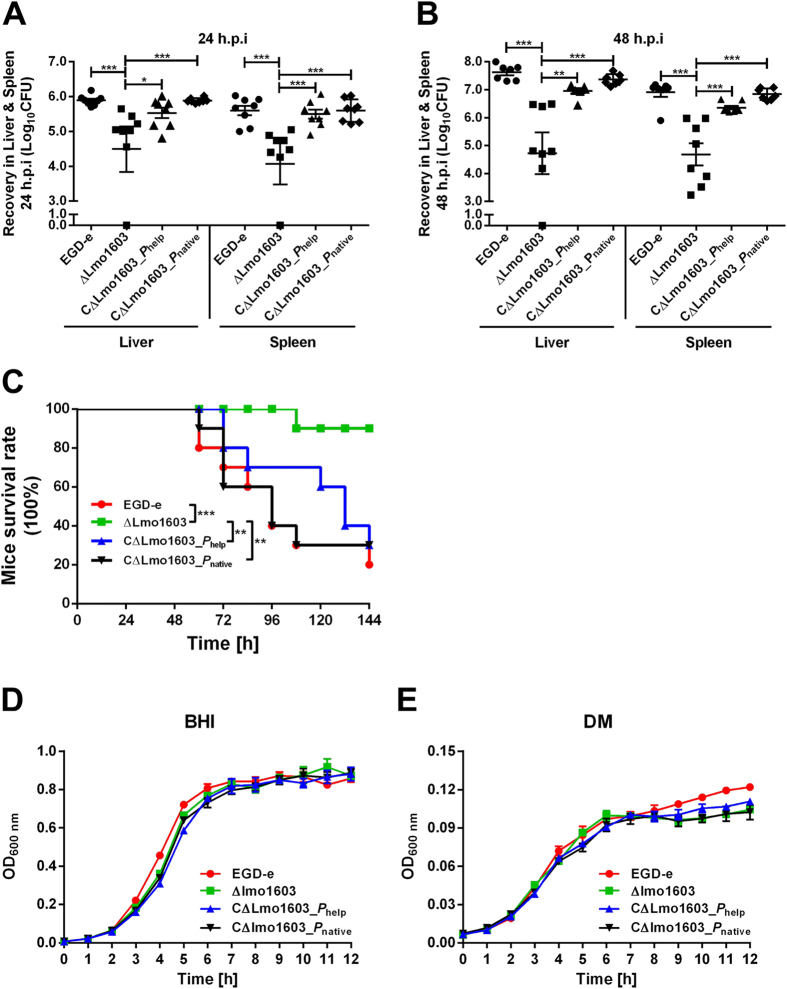
Lmo1603 is essential for L. monocytogenes virulence in mice, but not required for growth *in vitro*. The *L. monocytogenes* wild-type strain EGD-e, mutant ΔLmo1603 and two complemented strains CΔLmo1603_*P*_help_ and CΔLmo1603_*P*_native_ were inoculated i.p into ICR mice with 1 × 10^6^ CFU. Animals were euthanized 24 h **(A)** and 48 h **(B)** after infection and organs (liver and spleen) were recovered, homogenized, and homogenates serially diluted and plated on BHI agar. The bacterial number colonizing the liver and spleen is expressed as Mean ± SD of recovery Log_10_CFU per organ for each group. (**C**) Kaplan-Meier curve represents the survival of ICR mice over time. Ten mice in each experimental group infected i.p with 2 × 10^6^ cfu *L. monocytogenes* were monitored for up to 7 days after infection. Data is represented as percentage survival over time and significance was determined via a Log-rank test. *P < 0.05; **P < 0.01, ***P < 0.001. For *in vitro* growth assay, overnight cultures were re-suspended in fresh BHI (**D**) or defined medium (DM) (**E**), and incubated at 37 °C for 12 h. Kinetic growth was then measured (OD_600 nm_) at 1-h interval. The data are expressed as Mean ± SD of three independent experiments.

As part of an ongoing screen to determine the potential role of peptidases in *L. monocytogenes* pathogenesis, the virulence of Δ*lmo1603* mutant was evaluated in a murine model. The ICR mice were inoculated intraperitoneally with 10^6^ bacteria. Infected mice were euthanized 24 h and 48 h after infection, and the liver and spleen samples were recovered. Bacterial burden in these organs were significantly (***P***** <** **0.001**) fewer in the Lmo1603 deletion mutant than those in the wild-type strain 24 and 48 h post infection (Fig. 3A,B). Furthermore, inactivation of Lmo1603 resulted in 90% survival of the animals infected intraperitoneally with 10^6^ bacteria (Fig. 3C). In contrast, infection with the same number of the wild-type strain led to 80% mortality (***P***** <** **0.001**). To verify the roles of Lmo1603, we constructed two complemented strains, CΔLmo1603_*P*_help_ and CΔLmo1603_*P*_native_, expressing Lmo1603 under a constitutive promoter *P*_help_ and its native promoter *P*_native_, respectively. Impaired virulence of the mutant was fully rescued in the CΔLmo1603_*P*_native_ strain, but partly restored in the CΔLmo1603_*P*_help_ strain (Fig. 3A–C), suggesting that Lmo1603 was expressed *in vivo* in a precisely-regulated manner. To preclude the possibility that the survival phenotype of the Δ*lmo1603* mutant was not caused by growth defect in mice, growth of *Listeria* wild-type and mutant strains were performed in the rich medium (BHI) and a defined medium (DM). In both media, there were virtually no significant differences of growth among the strains (Fig. 3D,E), suggesting that this enzyme is not essential for growth in artificial media. Taken together, these demonstrate that the Lmo1603 deletion mutant was almost completely attenuated for virulence in mice.

### Lmo1603 is required for invasion to human intestinal epithelial cell Caco-2 and intracellular survival in murine macrophage RAW264.7.

**Figure 4:**
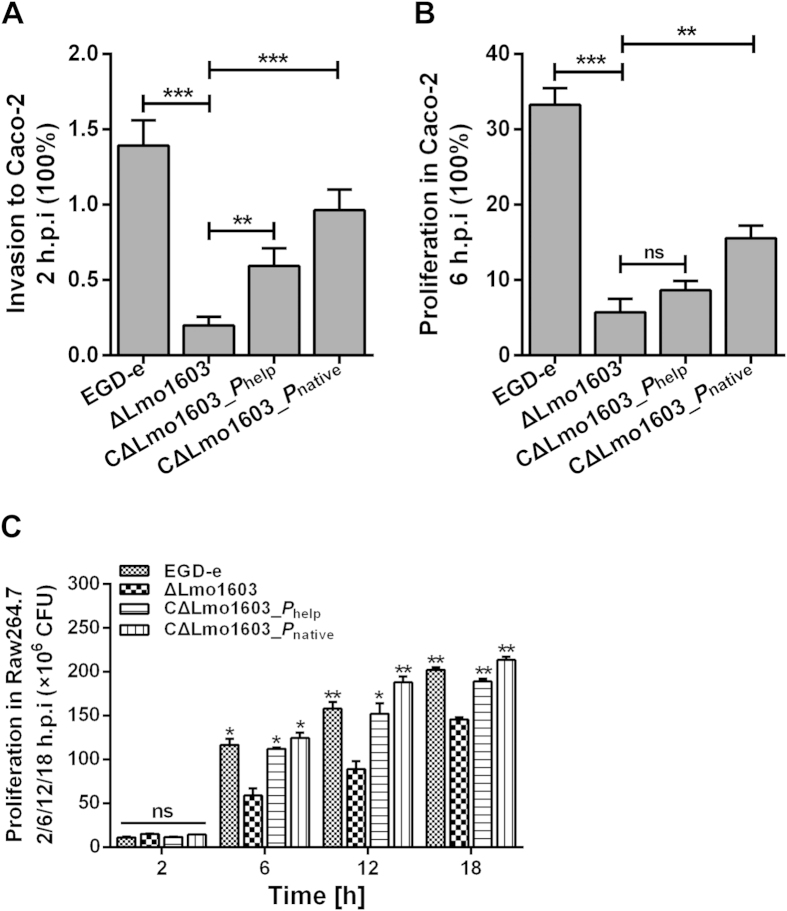
Lmo1603 is required for invasion into human intestinal epithelial cell Caco-2 and intracellular survival in murine macrophage RAW264.7. Caco-2 or RAW264.7 cells were infected with *L. monocytogenes* wild-type strain EGD-e, mutant ΔLmo1603 and two complemented strains CΔLmo1603_*P*_help_ and CΔLmo1603_*P*_native_ strains for one hour with multiplicity of infection (MOI) at 10:1. Gentamicin at 200 μg/ml was added to incubate for additional one hour to kill extracellular bacteria. At 2 h (**A**) and 6 h (**B**) post infection for Caco-2, or at 2, 6, 12 and 18 h (**C**) post infection for RAW264.7, the infected cells were lysed and serially diluted for enumeration on BHI agar plates. The number of bacteria able to invade and replicate in Caco-2 cells is expressed as Mean ± SD of recovery rate for each strain. The number of intracellular bacteria in RAW264.7 cells is expressed as Mean ± SD of CFU for each strain. **P < 0.01; ***P < 0.001, ns means no significance.

As presented above, Lmo1603 plays an important role in infection in mice. It is necessary to further explore whether this enzyme is required in infected human cells. Thus, invasion and proliferation assays in human Caco-2 and RAW264.7 cells were investigated. Cells were infected with the wild-type and mutant strains, and the number of intracellular bacteria was determined at 2 h and 6 h post-infection (hpi). Invasion of the Lmo1603 mutant into Caco-2 cells was significantly reduced (***P*** **<** **0.001**) (Fig. 4A), and its proliferation at 6 hpi was markedly impaired as compared to that of the wild-type strain (***P***** <** **0.001,** Fig. 4B). Such compromised cell invasion and proliferation ability of the mutant was almost completely restored in the CΔLmo1603_*P*_native_ strain, and partly in the CΔLmo1603_*P*_help_ strain (Fig. 4A,B). In murine macrophage RAW264.7, there was virtually no difference in intracellularly infected bacterial population at 2 hpi (Fig. 4C). However, survival of the mutant was markedly reduced in macrophages 6 hpi compared to wild-type and complemented strains (***P*** **<** **0.05)**, with the difference becoming more significant at 12 and 18 hpi (***P*** **<** **0.01)**. These results clearly indicate that Lmo1603 is required for *L. monocytogenes* invasion and survival in human intestinal epithelial cells and murine macrophages. Furthermore, this also strongly suggests that the virulence defect observed in murine infection model is likely to occur in human.

### Lmo1603 is present in the bacterial cytoplasm

**Figure 5:**
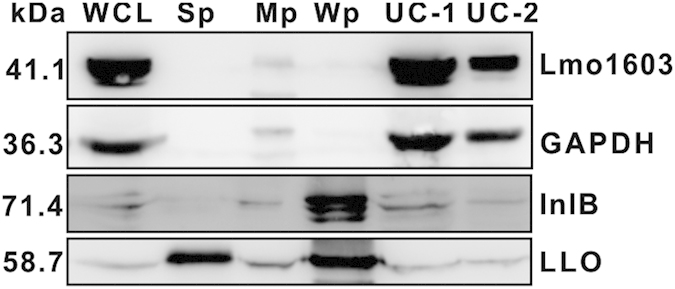
Lmo1603 is localized in the bacterial cytoplasm. Bacterial overnight cultures of the wild-type EGD-e were diluted (1:50) into 500 mL fresh BHI broth, and grown to the stationary phase. Bacterial pellets and culture supernatants were collected to obtain the different fractions of the cell according to the Materials and Methods (WCL: whole cell lysate; Sp: secreted protein; Mp: membrane protein; Wp: cell wall surface protein; UC-1: cytoplasm protein after 1^st^ round ultracentrifugation; UC-2: cytoplasm protein after 2^nd^ round ultracentrifugation). Proteins were separated through a 12% SDS PAGE and immunoblotted with α-Lmo1603, α-InlB, α-LLO or α-GAPDH antisera. The predicted molecular weight of each protein is indicated on the left.

Given the significant defect in virulence of the Lmo1603 mutant, it is critical to determine the localization of Lmo1603 in *L. monocytogenes.* Typically, aminopeptidases from other families are secreted and function extracellularly in a variety of bacterial species^10^,^27^,^28^. However, the protein localizations of M29 family members remain poorly understood. We prepared cell fractionations and identified that this enzyme is almost entirely located within the bacterial cytoplasm with only a very small fraction residing in the cell membrane (Fig. 5). In addition, Lmo1603 was not detected in secreted protein fractions (Fig. 5). Separation of cytoplasmic, secreted, membrane-associated and cell wall fractions was verified by immunoblotting with marker proteins: GAPDH (cytoplasm), LLO (secreted protein)^29^, and InlB (cell wall anchored protein)^30^. These strongly indicate that Lmo1603 is predominantly located inside bacterial cells, and its virulence impact is not from the extracellular milieu.

## Discussion

Aminopeptidases, widely distributed in nature, are a group of exopeptidases that have the ability to selectively release N-terminal amino acid residues from peptides and proteins^28^,^31^. The roles of aminopeptidases fall into one of three general categories: nitrogen metabolism, biosynthesis of active peptides, and protein activation/degradation^32^,^33^,^34^,^35^. Recently, the importance of these enzymes has been emphasized. Aminopeptidases play crucial roles in pathogenesis in a variety of bacterial and protozoan pathogens. Of particular interest is the M17 family leucine aminopeptidases which have been investigated in bacteria, in particular in Gram-negative bacteria with virulence association^36^,^37^,^38^. In addition, Luckett and his colleagues have demonstrated that the M28 family arginine-specific aminpeptidase acts as an important virulence factor in successful *Pseudomonas aeruginosa* infections^10^. For the M29 family aminopeptidases T, however, most of the studies have restricted to their biochemical properties and biophysical characterizations^16^,^18^,^19^,^39^. Here, we demonstrate that an M29 family aminopeptidase T (encoded by Lmo1603) from *L. monocytogenes* possesses a typical ability to catalyze the cleavage of amino acids from peptide substrates. Until now, the potential contribution of the M29 family members to bacterial virulence remains unknown. Apparently Lmo1603 of *L. monocytogenes*, though located inside the bacterial cytoplasm, affects virulence, which is in contrast to other virulence-associated aminopeptidases of other bacteria where the enzyme is secreted and is extracellularly functional^10^,^27^. In *Staphylococcus aureus*, an M17 family leucine aminopeptidase localized to the bacterial cytosol is required to modulate virulence via its aminopeptidase activity^36^. In *L. monocytogenes*, we propose a similar mechanism of action. Rather than direct processing of secreted virulence-related proteins, Lmo1603 most likely functions by modulating the key protein targets within the bacterial cells.

The M29 family encompasses AmpT, AmpII, PepS, and AmpS^11^. All these enzymes contain a highly conserved catalytic triad (Glu-Glu-His-Tyr-His-Asp), which plays significant roles in catalysis and substrate binding^16^,^17^. For AmpS from *Staphylococcus aureus* as an example, the active sites are located at opposite ends of a large internal cavity. Two metal ions with full occupancy and a third metal ion with low occupancy are present in the active site. A water molecule and Glu319 serve as bridging ligands to the two metals with full occupancy. One of these metal ions is additionally coordinated by Glu253 and His348 and the other by His381 and Asp383. In addition, the metals are involved in weak metal-donor interactions to a water molecule and to Tyr355^13^. For *L. monocytogenes*, Glu216, Glu281, His308, Tyr315, His217 and Asp329 are the corresponding active sites of Lmo1603, as confirmed by site-directed mutagenesis revealing that single mutation at any one of these residues completely abolished the enzymatic activity. Interestingly, Lmo1603 can be activated by Fe^3+^, Zn^2+^ and Mn^2+^, and is strongly stimulated by Co^2+^. In bacterial infection in the host, metal iron is essential for the host cells and the pathogen, as both require this metal as a cofactor for functional expression of many proteins or as a prosthetic group for essential enzymes that are involved in many basic cellular functions and metabolic pathways^40^,^41^. Important virulence factors of *L. monocytogenes* that facilitate bacterial intracellular invasion and spreading (including ActA, LLO and the regulator PrfA) are positively controlled by iron limitation^42^. As numerous metals in the host environment are available, but in low or trace quantities, bacteria have evolved active metal acquisition systems (metal ABC transporters) with high affinity to maintain iron equilibrium during infection. The opportunistic pathogen *Staphylococcus aureus* employs a cobalt transporter (Cnt) for ion uptake, which is required for full bacterial virulence and optimal colonization of the urinary tract in murine infection models^43^. For *L. monocytogenes*, there also exists a cobalt transporter homologue encoded by *lmo1207* in its genome^41^, which might work collaboratively with Lmo1603, a cobalt-dependent aminopeptidase, to maintain Co^2+^ homeostasis in favor of its infection.

Most importantly, we found that deletion of Lmo1603 led to a dramatic reduction in bacterial burden in the liver and spleen during systemic murine infection. *Pseudomonas aeruginosa* employs an arginine-specific aminopeptidase (belongs to M28 family) to provide a fitness advantage in environments where the sole source of nitrogen is peptides with an aminoterminal arginine, which could be important for establishing a successful infection^10^. *Porphyromonas gigivalis* relies on an arginine-specific peptidase to survive in anaerobic conditions where it causes infection and inflammation^44^. However, considering that there is no growth defect in the Lmo1603 deletion mutant that might account for decreased virulence to mice, we suggest that Lmo1603 plays a non-nutritional role during the infectious process. There is already a precedent for an intracellular M17 family leucine aminopeptidase that is required for virulence, but not for *in vitro* growth in *Staphylococcus aureus*^36^. For *L. monocytogenes*, the fact that Lmo1603 contributes to bacterial virulence might be attributed to the ability of this enzyme to preferably catalyze the peptides to release free arginine, which serves as a substrate for a variety of catabolic pathways^45^,^46^,^47^. In many microorganisms arginine can be used as a substrate for arginase pathway to catalyze arginine to urea and ornithine^47^. In host cells, arginine is also a substrate for the inducible nitric oxide synthase (iNOS). The iNOS enzyme combines arginine and oxygen to form nitric oxide which inhibits bacterial survival^10^. Elevated levels of arginine stimulate arginase activity in the bacterial cells, which will in turn inhibit inducible nitric oxide synthase (iNOS) response in macrophages by depletion of arginine and thus serves as a strategy for bacterial survival^47^,^48^. Alternatively, the loss of Lmo1603 influences virulence probably because one of its function is to degrade regulatory proteins and modify the expression of virulence-associated factors. As for *Salmonella*, for example, the Lon protease causes down-regulation of pathogenicity-related gene expression by degrading the HilC and HilD regulator proteins^49^,^50^. It is also possible that Lmo1603 may utilize its aminopeptidase activity to inactivate host proteins and thereby aid pathogenicity of *L. monocytogenes*. Such a mechanism has been previously reported to be employed by *Haemophilus influenza* for enhanced adherence to epithelial cells^51^. Furthermore, the role of Lmo1603 and its underlying mechanisms during the oral route of *L. monocytogenes* infection, which is more physiologically important and natural, needs to be investigated.

To our knowledge, it is the first time that an M29 family aminopeptidase T encoded by *Lmo1603* from *L. monocytogenes* has been characterized biochemically and functionally. We have demonstrated that *L. monocytogenes* Lmo1603 is not only involved in bacterial adhesion and invasion to host cells but also contributes to pathogenicity.

## Materials and Methods

### Bacterial strains, plasmids, and culture conditions

*Listeria monocytogenes* EGD-e strain was used as the wild-type. *Escherichia coli* DH5α was employed for cloning experiments and as the host strain for plasmids pET30a(+) (Merck, Darmstadt, Germany), pIMK2 and pKSV7. *Escherichia coli* Rosetta (DE3) (Merck) was used for protein prokaryotic expression. *L. monocytogenes* was cultured in brain heart infusion (BHI) medium (Oxoid, Hampshire, England). *E. coli* DH5α and Rosetta (DE3) were grown at 37°C in Luria-Bertani broth (LB) (Oxoid). Stock solutions of ampicillin (50 mg/ml), erythromycin (50 mg/ml), kanamycin (50 mg/ml), and chloramphenicol (50 mg/ml) were added to the media when necessary. All chemicals were obtained from Sangon Biotech (Shanghai, China), Merck, or Sigma-Aldrich (St. Louis, US) at the highest purity available.

### Bioinformatics analysis

Alignment of nucleotide and deduced amino acid sequences was performed with MUSCLE by using Geneious software^52^. Phylogenetic tree was constructed with Neighbor-joining (NJ) method using 100 bootstrap replicates. The amino acid sequences of Lmo1603 from *L. monocytogenes* EGD-e and its homologues in other microbial species were obtained from Genbank database (http://www.ncbi.nlm.nih.gov/) and aminopeptidase family membership interrogated via the MEROPS database (http://merops.sanger.ac.uk/)^11^. The known crystal structures of aminopeptidase were acquired from the Protein Data Bank (PDB, http://www.rcsb.org/pdb/home/home.do/). The putative model of Lmo1603 was constructed through the SWISS-MODEL Workspace^53^,^54^, using the aminopeptidase with known 3D structure as the template.

### DNA manipulations

Preparation of plasmids, DNA manipulations, and transformation of *E.coli* competent cells were performed as previously described^55^ and genomic DNA was extracted from *L. monocytogenes* according to the previous reports^45^,^56^. Oligonucleotide primers were synthesized by GENEWIZ Inc., (Suzhou, China). PCR fragments were purified using the AxyPrep DNA Gel Extraction Kit (Axygen Inc., US) and digested with defined restriction enzymes (NEB, Ipswich, US) to facilitate the insertion into vectors. Positive clones were then sequenced by GENEWIZ Inc. to verify presence of any target mutations.

### Construction of *lmo1603* deletion mutant

The temperature-sensitive pKSV7 shuttle vector was used for creating mutations from *L. monocytogenes* strain EGD-e background. Genomic DNA was extracted as described previously^56^,^57^. A homologous recombination strategy with SOE-PCR procedure was used for in-frame deletion to construct *lmo1603* deletion mutant^58^. Specifically, the DNA fragments containing homologous arms upstream and downstream of *lmo1603* were obtained by PCR amplification of EGD-e DNA templates using the SOE primer pairs Lmo1603-a/Lmo1603-b and Lmo1603-c/Lmo1603-d (Table S1). The obtained fragment was then cloned into the vector pKSV7 and electroporated into the competent EGD-e cells. Transformants were grown at a non-permissive temperature (41 ^o^C) in BHI medium containing chloramphenicol to promote chromosomal integration and the recombinants were passaged successively in BHI without antibiotics at a permissive temperature (30 ^o^C) to enable plasmid excision and curing^59^. The recombinants were identified as chloramphenicol-sensitive colonies and confirmed by PCR with primers Lmo1603-a-front (external to Lmo1603-a) and Lmo1603-d (Table S1). The resultant in-frame deletion mutant was further verified by DNA sequencing and finally designated as ΔLmo1603.

### Complementation of *lmo1603* deletion mutant

To complement the *L. monocytogenes* ΔLmo603 strain, we constructed two complemented strains by using the integrative plasmid pIMK2 which harbors a constitutive *Listeria* promoter *P*_help_^58^. For the first complemented strain, we amplified the complete ORF of *lmo1603* from EGD-e genomic DNA using the primer pairs Lmo1603-e/Lmo1603-f (Table S1) and inserted into the downstream of *P*_help_ after restriction with appropriate enzymes^58^. For the other one, the complete ORF of *lmo1603* along with its native promoter region was amplified using the primer pairs Lmo1603-g/Lmo1603-h (Table S1) and cloned into pIMK2 following restriction to cut off the *P*_help_ region with enzymes. The resulting plasmids were then respectively electroporated into the ΔLmo1603 strain. Regenerated cells were plated on BHI agar containing kanamycin (50 μg/ml). The two complemented strains were designated as CΔLmo1603_*P*_help_ and CΔLmo1603_*P*_native_, respectively.

### Prokaryotic expression and purification of recombinant Lmo1603

Lmo1603 was expressed as fusion protein to the N-terminal His-tag using pET30a(+) as the expression vector. *E. coli* Rosetta (DE3) was used as the expression host. The full-length ORF of *lmo1603* gene from EGD-e genome was amplified with the primer pairs Lmo1603-exp-F/R (Table S1) and then inserted into the vector. The resulting plasmid was designated as pET30a-Lmo1603. *E. coli* cells harboring the recombinant plasmids were grown in 500 ml LB medium supplemented with 50 μg/ml kanamycin at 37 °C  until the cultures reached 0.6–0.8 at OD_600nm_. Isopropyl β-D-1-thiogalactopyranoside (IPTG) was then added to a final concentration of 0.4 mM to induce expression of Lmo1603 for additional 4 h at 37 °C. The His-tagged soluble protein was purified using the nickel-chelated affinity column chromatography. Briefly, IPTG-induced cell pellets were collected, resuspended in 50 mM PBS (pH 7.4), and disrupted with sonication at 300w for 5s with intermittent cooling on ice for 10s (30 min in total). After centrifugation at 13,000 g for 30 min, the supernatant samples were collected and loaded onto a 2-ml prepacked nickelchelated agarose gel column (Weishi-Bohui Chromtotec Co., Beijing, China). The columns were washed with PBS containing 0.5 M NaCl and 50 mM imidazole, and the target protein was eluted with a linear gradient of 25–500 mM imidazole prepared in the same buffer. Expression and purification of the recombinant protein was analyzed by 12% SDS-PAGE followed by Coomassie Brilliant Blue staining. Protein concentration was quantified using the NanoDrop (Thermo Fisher Scientific, Lafayette, US) for the following polyclonal antibody preparation and aminopeptidase activity assay.

### Preparation of polyclonal antibodies against the recombinant proteins

The purified recombinant protein was used for raising polyclonal antibodies in New Zealand white rabbits according to a standard protocol^60^,^61^. Rabbit was first immunized by sub-cutaneous injections of 500 μg protein with equal volume of Freund’s complete adjuvant (Sigma-Aldrich, St. Louis, US). After two weeks, the rabbit was boosted subcutaneously three times with 250 μg protein each in incomplete Freund’s adjuvant (Sigma) at 10-day intervals. Rabbits were bled ~10 days after the last injection.

### Cell fractionation and protein localization of Lmo1603

Overnight bacterial cultures of the wild-type EGD-e were diluted (1:50) into 500 mL fresh BHI broth, and bacteria were grown to the stationary phase (OD_600nm_ reaches to 0.6–0.8). **For secreted proteins isolation:** The fractionation procedure was described by Lenz and Portnoy^62^, with minor modifications. Briefly, the bacterial cells were pelleted by centrifugation at 13,000 g for 20 min at 4 °C, and the resulting culture supernatant collected and filtered through a 0.22-μm polyethersulfone membrane filter (Millipore, Boston, US). Trichloroacetic acid (TCA) was added to the supernatant to reach a final concentration of 10%, left on ice overnight and washed with ice-cold acetone. The washed precipitates of supernatant proteins were re-suspended in SDS-PAGE sample buffer (5% SDS, 10% glycerol, and 50 mM Tris-HCl, pH 6.8). Samples were boiled for 5 min and stored at −20 °C before electrophoresis. **For total cell proteins isolation:** The previous method was applied^63^. Specifically, the bacterial pellets were re-suspended in 1 ml of extraction solution (2% Triton X-100, 1% SDS, 100 mM NaCl, 10 mM Tris-HCl, 1 mM EDTA, pH 8.0). One gram of glass beads (G8772, Sigma-Aldrich) was added and samples lysed by using the homogenizer Precelly 24 (Bertin, Provence, France) at 6,000 rpm for 30 s with intermittent cooling for 30 s (2 cycles in total) and then centrifuged at 12,000 rpm for 15 min. Supernatant was retained as the whole cell extract. **For membrane proteins extraction:** The whole cell extract was ultracentrifuged at 100,000 g for 1 h at 4 °C to pellet the membranes. The pellets were re-suspended in 1 ml extraction solution and ultracentrifuged at 100,000 g for an additional 1 h at 4 °C. The resulting supernatant fractions were removed and the pellets that represent the membrane-containing fraction were kept at −20 °C  before use. **For cell wall proteins isolation:** The procedure was described before^30^, with minor modifications. Briefly, the bacterial pellets were re-suspended in approximately 0.5% of the original culture volume of PBS containing 2% (w/v) SDS for 30 min at 37 °C with gentle shaking. Bacterial suspensions were centrifuged at 12,000 g for 10 min, the supernatant contained the extracted cell wall proteins. Finally, the extract was filtered through a 0.22-μm filter and the filtrate was ready to be analyzed by SDS-PAGE or by immunoblotting.

### Enzyme activity assay and substrate specificity analysis

Aminopeptidase activity was determined spectrophotometrically in the assay buffer (50 mM Tris-HCl, pH 8.5) containing 2 mM amino acid-*p*-nitroanilide (XXX-*p*NA) substrate. Absorbance at 405nm, due to the release of *p*-nitroaniline, was monitored at 1-h intervals for 12 h by using Micro-plate reader Synergy H1 (BioTek Solutions, Inc., Santa Barbara, US)^27^. The substrate-specificity of purified Lmo1603 was assayed against several amino acid-*p*NA substrates at the same concentration of 2 mM (Leu-*p*NA, Ala-*p*NA, and Arg-*p*NA).

### Effects of metal ions on Lmo1603 aminopeptidase activity

To determine the effect of metal ions on Lmo1603 aminopeptidase activity, the purified enzyme with 1 mM Co^2+^, Mg^2+^, Fe^3+^, Zn^2+^, Mn^2+^or Ni^2+^ was incubated at 37 °C for 1 h, and enzyme activity was measured in 50 mM Tris–HCl (pH 8.5) using Arg-pNA as the substrate. The relative enzyme activity was calculated from control samples (without addition of any metal ion) set to 100%.

### Site-directed mutagenesis

To identify the predicted active sites of Lmo1603, single site-directed mutants (E216A, E281A, H308A, Y315A, H327A and D329A) were generated on the original vector template pET30a-Lmo1603 using the QuickChange Site-Directed Mutagenesis Kit (Agilent, Santa Clara, USA) with the oligonucleotide primer pairs (Table S1). Template DNA was then removed by digestion with Dpn I (TOYOBO, Osaka, Japan) for 2 h at 37 °C . All mutants were sequenced to ensure that only the desired single mutations had been incorporated correctly into the wild-type expression construct. The corresponding mutant proteins were designated as E216A, E281A, H308A, Y315A, H327A and D329A accordingly. Their expression, purification and enzymatic assay were performed as described above for the wild-type protein.

### Growth analysis in BHI broth and defined medium (DM)

*L. monocytogenes* wild-type strain EGD-e, mutant ΔLmo1603, and two complemented strains, CΔLmo1603_*P*_help_ and CΔLmo1603_*P*_native_ were grown overnight at 37 °C in BHI broth with shaking. The cultures were collected by centrifugation at 5000 × g at 4 °C, washed once in PBS (10 mM, pH 7.4). The initial OD_600 nm_ of each bacterial suspension was adjusted to 0.6. The bacteria were then 1:100 diluted in fresh BHI broth or defined medium (DM, prepared according to Phan-Thanh *et al.*^64^), and incubated at 37 °C  for 12 h. Kinetic growth was measured (OD_600 nm_) at 1-h interval.

### Proliferation in human intestinal epithelial cell Caco-2 and murine macrophage RAW264.7

Bacterial survival or proliferation in human intestinal epithelial Caco-2 cells and murine macrophage cells RAW264.7 was determined as previously described^45^,^65^. Stationary cultures of the wild-type EGD-e, mutant ΔLmo1603, and two complemented strains at 37 °C in BHI were washed and re-suspended in PBS (10 mM, pH 7.4). Caco-2 or RAW264.7 cells, cultured to 80% confluence in Dulbecco’s modified eagle medium (DMEM) containing 10% fetal calf serum were infected with the above strains for 60 min with multiplicity of infection (MOI) at 10:1. The culture supernatant was removed and the infected cells were treated with DMEM containing gentamicin at 200 μg/ml for 60 min to kill extracellular bacteria. The infected cells were lysed by adding 1 ml of ice-cold sterile distilled water at the indicated time points. The lysates were 10-fold diluted for enumeration of viable bacteria on BHI agar plates, which were considered as the two-hour numbers invading into the cells. The remaining cells were subjected to further incubation for 6, 12, and 18 h in 5% CO_2_ at 37 °C. Viable bacteria were enumerated as described above.

### Mice infection

All experiments were performed in accordance with guidelines and regulations as outlined and approved by the Laboratory Animal Management Committee of Zhejiang A&F University. The *L. monocytogenes* wild-type strain EGD-e, mutant ΔLmo1603, and two complemented strains were tested for their virulence to ICR mice (female, 18–22 g, purchased from Zhejiang Academy of Medical Sciences, Hangzhou, China) and recovery in their liver and spleen samples as described previously^66^. The mice (8 per group) were injected intraperitoneally with about 1 × 10^6^ cfu of each strain. At 24 and 48 h post-infection, mice were euthanized and their liver and spleen were removed and individually homogenized in PBS (10 mM, pH 7.4). Surviving listerial cells were enumerated by plating serial dilutions of the homogenates on BHI agar plates. The results were expressed as Mean ± SD of recovery bacterial number (Log_10_CFU) per organ for each group. For animal survival experiments, mice injected intraperitoneally with 2 × 10^6^ cfu listeria were monitored for up to 7 days after infection. Survival curves were calculated by using the Kaplan-Meier method and differences in survival were determined by using the Log-rank test.

### Statistical analysis

Data were analyzed using the two-tailed homoscedastic Student’s *t*-test. Differences with *P* values < 0.05 were considered as statistically significant.

## Additional Information

**How to cite this article**: Cheng, C. *et al.* Aminopeptidase T of M29 Family Acts as A Novel Intracellular Virulence Factor for *Listeria monocytogenes* Infection. *Sci. Rep.* **5**, 17370; doi: 10.1038/srep17370 (2015).

## Supplementary Material

Supplementary Information
