# Supplementary material for: Aminopeptidase T of M29 Family Acts as A Novel Intracellular Virulence Factor for Listeria monocytogenes Infection
Source: Sci Rep. 2015 Nov 27;5:17370. doi: 10.1038/srep17370 (PMC4661694; doi:10.1038/srep17370)
Supplement: Supplementary Information [file srep17370-s1.pdf]

Aminopeptidase T of M29 Family Acts as A Novel Intracellular Virulence Factor for *Listeria monocytogenes* Infection

Changyong Cheng[1]*, Xiaowen Wang[1]*, Zhimei Dong[1], Chunyan Shao[1], Yongchun Yang[1], Weihuan Fang[1,2], Chun Fang[2], Hang Wang[1], Menghua Yang[1], Lingli Jiang[3], Xiangyang Zhou[3], Houhui Song[1#]

Table S1. PCR primers used in this study. Nucleotides introduced to create restriction enzyme sites are underlined. All primers were synthesized by GENEWIZ, Inc., Suzhou, China.

Primer name	Primer sequence (5'-3')	Product (bp)	Reference
Lmo1603-a	CCGGAATTCAATGTCACCAACTGCATCAAAAACTG	534	This study
Lmo1603-b	AAGCGCCTTTTTATTGTAACCTCCCTAAATTTTAACTAAAAATAGG		This study
Lmo1603-c	GGAGGTTACAATAAAAAGGCGCTTAGGACAGAATCCT	585	This study
Lmo1603-d	TGGCTGCAGACACAAGCTCCAAGATTTGAAATGGA		This study
Lmo1603-a-front	AGAAAGAATTGAATTGCTAATTGAGACAGC		This study
Lmo1603-e	CGCGGATCCTATGAGAGATGCAAGAATCGAAAAATTAG	1132	This study
Lmo1603-f	CCGCTCGAGTTATACTAAGTTTTCTGGATTAAGTGCTTCTAATT		This study
Lmo1603-g	GACGAGCTCCTATCCACACCTCCATATTCCTTTTTC	1386	This study
Lmo1603-h	CCGCTCGAGTTATACTAAGTTTTCTGGATTAAGTGCTTCTAATT		This study
Lmo1603-exp-F	GCGCATATGAGAGATGCAAGAATCGAAAAATTAGCA	1125	This study
Lmo1603-exp-R	CCGCTCGAGTACTAAGTTTTCTGGATTAAGTGCTTCTAATTCT		This study
Lmo1603_E216A-F	AATCACGAACTGGCGCAGTAAATTGCTCACCGTCTGGAATATTCATTTC	6348	This study
Lmo1603_E216A-R	GAAATGAATATTCCAGACGGTGAGCAATTTACTGCGCCAGTTCGTGATT		This study
Lmo1603_E281A-F	ACCCCAATAGCGAATGCACCAACGAAGCGCG	6348	This study
Lmo1603_E281A-R	CGCGCTTCGTTGGTGCATTCGCTATTGGGGT		This study

Lmo1603_H308A-F	CATAACATTGACCAGGCGTAAAGGCGAAACTACCTTCAATTTTTTCG	6348	This study
Lmo1603_H308A-R	CGAAAAAATTGAAGGTAGTTTCGCCTTTACGCCTGGTCAATGTTATG		This study
Lmo1603_Y315A-F	GTTGCCATTAAATGCTTCGTCAGCACATTGACCAGGCGTAAAGTG	6348	This study
Lmo1603_Y315A -R	CACTTTACGCCTGGTCAATGTGCTGACGAAGCATTTAATGGCAAC		This study
Lmo1603_H327A-F	TGTTAACTAAATCCCAGGCAATAGCAGATTGGTTGCCATTAAATGCTTCG	6348	This study
Lmo1603_H327A-R	CGAAGCATTTAATGGCAACCAATCTGCTATTGCCTGGGATTTAGTTAACA		This study
Lmo1603_D329A-F	CACGTTGAATGTTAACTAAAGCCCAGTGAATAGCAGATTGG	6348	This study
Lmo1603_D329A-R	CCAATCTGCTATTCACTGGGCTTTAGTTAACATTCAACGTG		This study

Figure S1

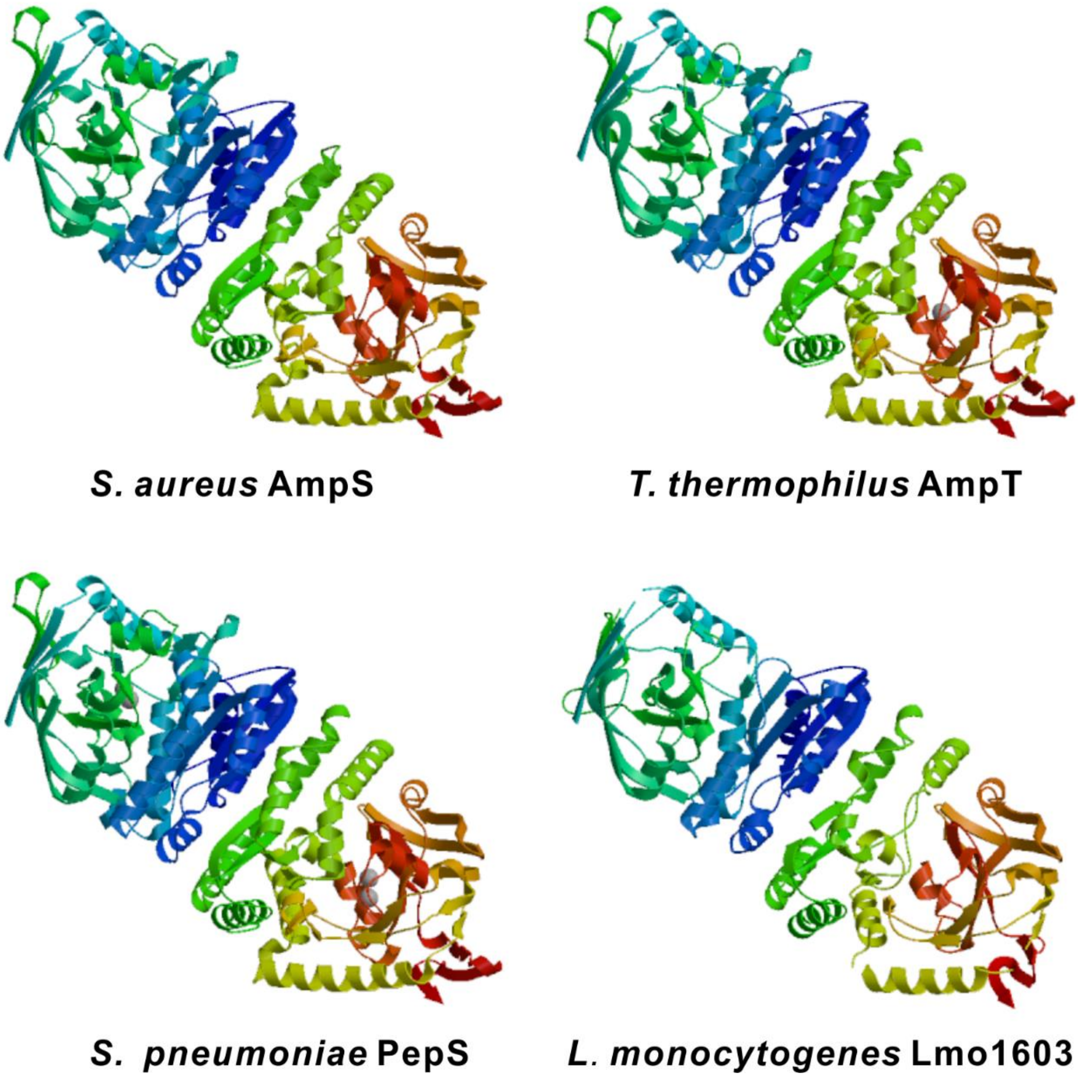

Figure legends

Figure S1. Predicted protein structure of *L. monocytogenes* Lmo1603. The putative crystal models of Lmo1603 constructed with SWISS-MODEL Workspace using the known crystal structures of the members of M29 family as the templates. These templates are *Staphylococcus aureus* AmpS (PDB: 1ZJC), *Thermus thermophilus* AmpT (PDB: 2AYI) and *Streptococcus pneumoniae* PepS (PDB: 4ICQ).
